# A Lightweight Framework for Protected Vegetable Disease Detection in Complex Scenes

**DOI:** 10.1002/fsn3.70200

**Published:** 2025-05-03

**Authors:** Jun Liu, Xuewei Wang, Qian Chen

**Affiliations:** ^1^ Shandong Provincial University Laboratory for Protected Horticulture Weifang University of Science and Technology Weifang China; ^2^ School of Computer, Sichuan Technology and Business University, Chengdu China

**Keywords:** adaptive feature enhancement, deformable attention mechanism, lightweight object detection, protected vegetable diseases, transfer learning

## Abstract

The rapid development of computer vision technology has provided new technical support for smart agriculture. Vegetable diseases represent a significant threat to agricultural production, with severity that cannot be ignored. However, through scientifically effective prevention and control measures, these negative impacts can be significantly mitigated. Intelligent disease detection systems, as advanced methods replacing traditional manual inspection, have become important means for developing smart agriculture and improving the efficiency of vegetable production management. Nevertheless, traditional manual detection is not only time‐consuming and labor‐intensive but also faces accuracy limitations, while existing computer vision detection methods still encounter a series of challenges when confronting complex backgrounds, diverse disease manifestations, and varying degrees of occlusion in real cultivation environments, including insufficient anti‐interference capabilities, limited detection precision, and suboptimal real‐time performance. This research addresses the practical challenges of limited data acquisition and sample scarcity for protected vegetable diseases by proposing an innovative strategy that implements differentiated data augmentation technique combinations for different categories of samples, significantly enhancing the model's resistance to environmental interference. Based on the integrated concepts of machine vision and deep learning, we developed a lightweight vegetable disease detection network named VegetableDet. This network innovatively combines Deformable Attention Transformer (DAT) with YOLOv8n backbone architecture, enhancing perception capabilities for long‐range feature dependencies. Simultaneously, a Channel‐Spatial Adaptive Attention Mechanism (CSAAM) is integrated into the Neck network, achieving precise localization and enhancement of key features. To address the issue of low model convergence efficiency, we further designed a hierarchical progressive transfer learning training strategy, effectively accelerating the model adaptation process and improving detection accuracy. Experimental evaluation demonstrates that on our custom comprehensive protected vegetable disease dataset, the VegetableDet model exhibits excellent performance in detecting 30 diseases and healthy samples across 5 vegetable types, with precision (P), recall (R), and average precision (AP) all exceeding 90%, and an overall mean Average Precision (mAP) reaching 94.31%. The model demonstrates powerful adaptability under complex environmental conditions, providing reliable technical support for real‐time monitoring and precise prevention and control of protected vegetable diseases, with broad application prospects.

## Graphical abstract

VegetableDet is an innovative lightweight framework for protected vegetable disease detection, integrating Deformable Attention Transformer (DAT) and Channel‐Spatial Adaptive Attention Mechanism (CSAAM) to effectively address detection challenges in complex environments. Experiments demonstrate that the model achieves 94.31% mean Average Precision (mAP) in detecting 30 diseases across 5 vegetables, with only 2.83 M parameters, fast inference speed, and strong environmental adaptability.

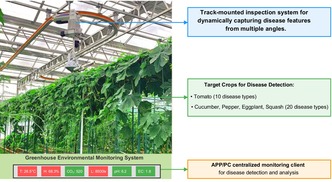

## 1 Introduction

With the continuous development of computer vision technology, its applications in practical scenarios have become increasingly widespread. Vegetable diseases represent a major threat, with severity comparable to natural disasters affecting humans. However, unlike natural disasters, the impact of vegetable diseases can be effectively alleviated through scientific prevention and control measures (Liu, Zhu, et al. 2021). Replacing manual visual inspection with intelligent disease detection is crucial for advancing smart agriculture and vegetable production management, which has become a key task facing farmers. Traditional manual disease detection methods are both time‐consuming and labor‐intensive, making effective management difficult for farmers. Consequently, computer vision methods are increasingly being applied to protected vegetable disease detection in controlled environments (Upadhyay et al. 2025).

Cultivation of protected vegetables typically occurs in controlled environments such as greenhouses, which, while regulating temperature, humidity, and carbon dioxide concentration to promote plant growth, also create favorable conditions for pathogen reproduction, especially in high‐temperature and high‐humidity environments (Friha et al. 2021). Protected vegetable diseases can lead to dramatic yield reductions, with severe infections potentially causing total crop loss. Although the occurrence of vegetable diseases follows certain spatiotemporal patterns, their randomness and suddenness make prevention, diagnosis, and treatment complex (Zhang et al. 2024). Different vegetables are susceptible to different types of diseases, each with unique characteristics, making management more challenging. Misdiagnosis or delayed treatment can result in enormous losses, seriously affecting economics and livelihoods (Lin et al. 2024).

**FIGURE 1:**
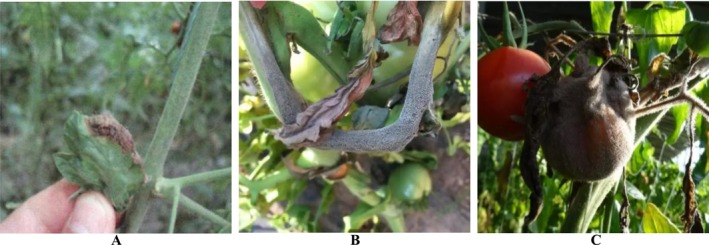
Early (A), Middle (B), and Late (C) Stage of Protected Vegetable Diseases.

In actual cultivation scenarios, detecting vegetable diseases faces numerous challenges, including varying sizes of infected areas, occlusion issues, and complex background environments. Existing detection algorithms often demonstrate poor robustness, low accuracy, and insufficient real‐time performance under these conditions (Zhang et al. 2021). An important characteristic of protected vegetable diseases is their dynamic development process. The location and size distribution of protected vegetable diseases exhibit high randomness and uncertainty. Unlike conventional computer vision tasks where object sizes are relatively fixed and only scale proportionally with shooting distance, protected vegetable diseases show significant scale variations during their development process. As shown in Figure 1, diseases initially appear as small lesions on leaves (Figure 1A), then rapidly expand to occupy most of the leaf area (Figure 1B), and may eventually spread to the entire plant (Figure 1C). This rapid transition process from minute lesions to large‐area infections makes it difficult for the same model to effectively identify and locate diseases at different developmental stages, with detection of early minor symptoms being particularly challenging.

Furthermore, the dense planting methods of protected vegetables further increase the complexity of disease detection. Factors such as mutual occlusion between plants, leaf overlap, and uneven lighting all affect the detection performance of models. Especially under conditions of leaf overlap or backlighting, disease characteristics can easily be obscured, leading to missed detections or false positives (Yan and Yang 2022). Meanwhile, the similar symptoms between different diseases also increase the difficulty of accurate diagnosis; for example, chlorosis phenomena may be caused by multiple pathogens or environmental factors, requiring more refined feature differentiation (Kang et al. 2024). Dai and Fan (2022) pointed out that industrial‐grade crop disease detection solutions need to simultaneously consider recognition time and accuracy, balancing performance and efficiency in actual agricultural scenarios.

Ferrag et al. (2022) emphasized the key role of deep learning and computer vision in Agriculture 4.0, particularly noting that applications in disease detection need to comprehensively consider Internet of Things (IoT) technologies and security performance to build complete intelligent agricultural systems. Protected vegetable disease detection systems, as important components of intelligent agriculture, need to efficiently integrate with other systems to achieve full‐process management from detection to prevention and control (Yang et al. 2021). Wang et al. (2022) proposed that under the background of Industry 5.0, parallel agricultural models based on cyber‐physical‐social systems will become future development trends, requiring disease detection technologies to adapt to the needs of this new agricultural production mode.

Currently, deep learning has become the focus of plant disease detection research because it can minimize bias in manual selection of disease features (Paul et al. 2024). In particular, Convolutional Neural Networks (CNNs) have demonstrated powerful capabilities in image classification and object detection tasks, able to automatically extract effective features from images. However, traditional CNN models typically have massive parameter counts and high computational complexity, making them difficult to deploy on resource‐constrained edge devices, which are common terminals in smart agricultural scenarios (Mhala et al. 2025). Additionally, CNN models primarily focus on local features of images, tending to ignore global contextual information, leading to decreased detection accuracy in complex backgrounds (Bonora et al. 2021).

Liu, Min, et al. (2021) significantly improved the accuracy of plant disease recognition by constructing a large‐scale plant disease dataset and proposing a visual region and loss reweighting approach, demonstrating the potential of deep learning in solving problems of random distribution, diverse symptoms, and complex backgrounds of plant diseases. However, building general models for specific vegetable disease types still faces challenges, especially in handling unseen disease types, where generalization ability needs improvement (Barbedo 2019). Barbedo (2018) research showed that factors affecting deep learning for plant disease recognition include dataset size, background complexity, symptom diversity, and image quality, requiring comprehensive consideration from multiple aspects to improve model effectiveness.

Zhao et al. (2021) effectively solved the data imbalance problem by using a Double Generative Adversarial Network (DoubleGAN) to generate unhealthy plant leaf images, which is significant for handling rare disease types. Particularly for multiple coexisting diseases in protected vegetables, generative models can help create more diverse training samples (Rahman et al. 2020). Hu et al. (2020) successfully improved the accuracy of pine disease recognition in UAV images by combining deep convolutional neural networks, deep convolutional generative adversarial networks, and AdaBoost classifiers, demonstrating the effectiveness of multi‐model fusion strategies in complex backgrounds.

Although advanced object detection networks like YOLOv8 gradually expand the receptive field through multi‐layer convolution operations, the relatively small convolution kernels in each layer make it easy to lose long‐range feature dependencies during feature extraction, which is crucial for accurately recognizing vegetable diseases at different development stages. Especially in dense planting environments, disease symptoms may be distributed across different parts of plants, requiring the model to have the ability to capture distant feature associations (Cheemaladinne and Srinivasa Reddy 2024). Wu et al. (2020) proposed combining the Deeplab V3+ semantic segmentation model with a phase‐based video magnification algorithm, successfully achieving respiratory rate detection for cattle, providing a new approach for protected vegetable disease detection by combining technologies from different domains.

In recent years, the Transformer architecture has made significant advances in the field of computer vision thanks to its powerful self‐attention mechanism, effectively modeling long‐range dependencies and capturing global contextual information (Wójcik Gront et al. 2024). Vision Transformer (ViT) demonstrates powerful image understanding capabilities by dividing images into fixed‐size patches and calculating relationships between patches (Sun et al. 2025). However, standard Vision Transformers tend to overfit when processing small to medium‐scale datasets, and their computational complexity increases quadratically with image size, limiting their application in resource‐constrained scenarios. Toda and Okura (2019) revealed the internal mechanism of convolutional neural networks diagnosing plant diseases through visualization methods, finding that neural networks can capture specific color and texture features of diseases, similar to human decision‐making processes, providing important references for interpretable artificial intelligence in agricultural applications.

Zhuang and Zhang (2019) achieved good results in recognizing the health status of broiler chickens, demonstrating the applicability of deep learning in animal health monitoring, which is equally important for early detection and warning of protected vegetable diseases. Wang et al. (2023) research on the application of infrared thermography and machine learning techniques in cattle health assessment showed that non‐contact detection methods combined with machine learning can effectively indicate changes in thermal biological properties in animal metabolism, providing new ideas for non‐destructive detection of protected vegetable diseases.

To address these issues, researchers have proposed various improved Transformer architectures. Among them, Vision Transformer with Deformable Attention (DAT) shows particular advantages by selecting the relative positions of keys and values in the attention mechanism in a data‐dependent manner, flexibly focusing on relevant regions and capturing more useful feature information (Xia et al. 2022). Compared to fixed‐window attention mechanisms, deformable attention can adaptively adjust the range and shape of attention based on input content, better adapting to disease features of different sizes and morphologies (Zhu, Su, Lu, Li, et al. 2020). Su et al. (2020) monitored wheat stripe rust in smart agriculture using aerial visual perception, combining UAV sensing, multispectral imaging, and deep learning U‐Net, proving that methods utilizing both spectral and spatial information outperform pure spectral classifiers, providing a multi‐modal fusion approach for protected vegetable disease detection.

Jelali (2022) conducted a comprehensive review of deep neural networks for tomato disease and pest detection using real field datasets, pointing out that most studies used the PlantVillage dataset collected under controlled conditions, leading to overly optimistic results, while there were fewer studies evaluating performance in complex backgrounds. This finding emphasizes the importance of evaluating model performance in actual facility environments. Jian et al. (2025) proposed a tomato leaf disease recognition method based on DGP‐SNNet, effectively solving the similarity problem between disease categories through hierarchical progressive transfer learning and partial convolution modules, which have important reference value for distinguishing multiple types of diseases in protected vegetables.

Li et al. (2024) proposed the YOLO‐Leaf model, utilizing dynamic snake convolution (DSConv) for feature extraction, improving the accuracy of apple leaf disease detection, and demonstrating the potential of special convolutional structures in plant disease detection. Mathew and Mahesh (2021) used YOLOv5 to detect bacterial spot disease in pepper plants, capable of detecting small lesions on leaves at a comparable speed and accuracy, which is significant for the early diagnosis of vegetable diseases. Liu, Lin, et al. (2021) proposed a CNN‐based algorithm that effectively identified three different cucumber leaf diseases, demonstrating the effectiveness of deep learning in detecting specific vegetable diseases.

To address the difficulties of vegetable disease data collection and limited sample availability, researchers have attempted various data augmentation and transfer learning techniques. Data augmentation creates new training samples by transforming original images, increasing data diversity and improving the model's generalization ability (Lin et al. 2024). Transfer learning helps models quickly adapt and converge on small‐scale target tasks by utilizing knowledge from models pre‐trained on large‐scale datasets (Attri et al. 2025). Kumar et al. (2021) proposed a rice leaf disease detection method based on a bidirectional feature attention pyramid network, achieving good results in combination with the YOLOv5 model, effectively addressing the detection problem of diseases at different scales through multi‐scale feature extraction.

Nagasubramanian et al. (2021) proposed a crop disease monitoring system based on ensemble classification and IoT, combining SVM and CNN to achieve continuous observation of plant growth and leaf diseases, providing timely decision support for farmers. Xin and Wang (2021) research on crop disease and pest image recognition based on deep learning showed that the DCNN‐G model integrated with Google data analysis performed excellently in disease recognition, while emphasizing the important impact of data quality on model performance. Lin et al. (2023) designed the TSBA‐YOLO tea leaf disease detection model based on a bidirectional feature attention pyramid network, enhancing the ability to acquire global information through a self‐attention mechanism, while the BiFPN feature fusion network and adaptive spatial feature fusion techniques improved the multi‐scale feature fusion capability of tea leaf diseases, enhancing the model's resistance to complex backgrounds.

Bao et al. (2023) research on UAV remote sensing detection of tea leaf blight based on DDMA‐YOLO proposed an effective monitoring method, reconstructing high‐resolution tea leaf images through a super‐resolution convolutional neural network (RCAN) to solve the problem of insufficient resolution in UAV remote sensing images, and adopting Retinex to enhance image contrast, reducing the impact of uneven lighting. Tian et al. (2023) proposed the VMF‐SSD method, enhancing texture feature information through multi‐scale feature fusion and a V‐space‐based position branch, effectively improving the performance of apple leaf disease detection, especially for lesions of different sizes.

Tanveer et al. (2025) proposed a two‐step feature extraction process combining VGG‐16 network and Gaussian Naive Bayes model, successfully improving the detection accuracy of maize crop health status. However, how to effectively implement knowledge transfer in the specific scenario of protected vegetable diseases remains a challenge to be solved. Zhou et al. (2023) demonstrated the possibility of maintaining high performance while reducing annotation work through combined supervised and weakly supervised deep learning methods for phenotypic analysis of plant leaf diseases, providing new approaches for the construction and utilization of protected vegetable disease datasets.

Addressing the above issues, this research proposes an innovative network framework specifically for protected vegetable disease recognition. Based on machine vision and artificial intelligence technologies, the following strategies are adopted to address the challenges of protected vegetable disease detection:

1. For the issue of long‐distance feature dependency relationships in vegetable diseases, the Deformable Attention mechanism (Vision Transformer with Deformable Attention, DAT) is introduced into the backbone network. The DAT module includes four key stages: first, embedding is performed through a 4 × 4 non‐overlapping convolution with a stride of 4; in stages 1 and 2, window local attention mechanisms are used to extract local features, utilizing the shifted window attention mechanism from Swin Transformer to move and integrate the calculation results of local attention within windows; in stages 3 and 4, feature maps continue to be processed through window local attention mechanisms to concentrate local information, while using deformable attention mechanisms to simulate global relationships between local information, flexibly adjusting the range and shape of attention, achieving effective alternation between local and global receptive fields, enabling the model to focus on long‐distance feature relationships while improving computational efficiency.
2. To address the diversity and complexity of disease manifestations, a lightweight adaptive attention mechanism (CSAAM) is introduced into the neck network, which can adaptively adjust weights in channel and spatial dimensions, highlighting key disease features, suppressing background interference, and improving the diversity and discriminative ability of feature expressions. The CSAAM module combines channel attention and spatial attention, capturing both “what is important” and “where is important” information, providing flexible and powerful representation capabilities for processing diseases of different sizes and morphologies.
3. To solve the significant disparity between the source domain (COCO dataset) and the target domain (vegetable disease image dataset), a hierarchical progressive transfer learning strategy is proposed. This strategy first pre‐trains the model on a tomato disease dataset, allowing it to acquire basic knowledge of vegetable diseases; subsequently, the pre‐trained parameters are transferred to the target task and further fine‐tuned on a comprehensive vegetable disease dataset, achieving effective knowledge transfer. This hierarchical transfer learning method fully utilizes the feature representation capabilities of pre‐trained models, accelerating model convergence and improving detection accuracy.
4. To address the difficulties of data collection and sample imbalance, appropriate proportions of data augmentation techniques are applied to different sample categories, balancing the distribution of data across categories and enhancing the model's learning capability. Simultaneously, Mosaic data augmentation technique is used during the training process, creating new images by splicing four different images together, further increasing the diversity and complexity of training samples to enhance the model's generalization performance.

Experimental results show that on the self‐built protected vegetable disease database, the proposed model achieves over 90% accuracy for 30 diseases across 5 vegetables, with an average precision (mAP) of 94.31%. Compared to other algorithms, the model significantly improves detection performance, with mAP increasing by 7.78% to 11.90%, and an inference time of only 23 ms. Even under challenging conditions such as lighting variations, complex occlusions, and background interference, the model maintains stable detection performance, demonstrating strong environmental adaptability and real‐time processing capabilities, providing effective technical support for intelligent monitoring and precise prevention and control of protected vegetable diseases.

## 2 Materials

### 2.1 Experimental Data Collection

**FIGURE 2:**
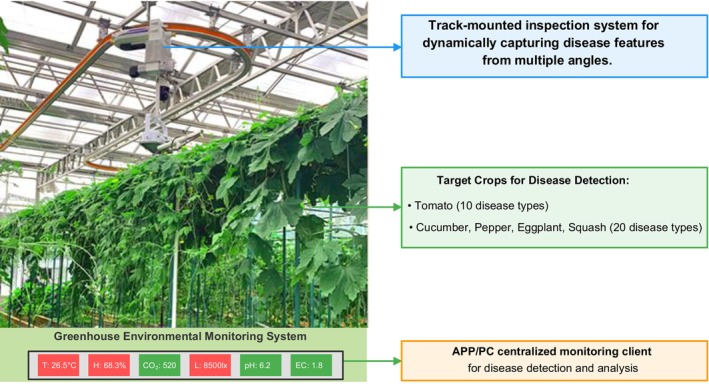
Schematic Diagram of Data Collection Sites.

In protected vegetable cultivation environments, the complex backgrounds of disease images present challenges for object detection tasks. Currently, existing datasets either only contain images for disease image classification tasks without object annotations, or only include disease images with object annotations captured in simple backgrounds. There is no publicly available effective object detection dataset specifically targeting real protected vegetable cultivation scenarios. Based on this, this research combined the experience of vegetable experts and used agricultural IoT monitoring equipment to collect disease images under different backgrounds (plant occlusion, leaf overlap, and soil interference) and lighting conditions (cloudy, sunny, dusk, night) from 42 greenhouse tunnels in 3 self‐built vegetable bases (covering 164 mu) of Weifang University of Science and Technology. The different collection conditions ensured the diversity of the dataset. A schematic diagram of the data collection sites is shown in Figure 2. A total of 35,986 protected vegetable disease samples were collected, including healthy and potentially infected images.

### 2.2 Data Preprocessing

The experimental images were all collected in protected vegetable cultivation environments. Due to the complexity and uncertainty of the field environment, the collected images may have issues such as object occlusion, overexposure, poor focus, and target deviation, requiring preprocessing of the original image dataset. The first step involved screening the collected images through data cleaning to remove ineffective image data with high similarity, low resolution, or poor clarity, retaining images with better imaging effects. The second step involved cropping the obtained image data to remove some redundant information in the images while retaining regions of interest with disease spots to improve image information quality. The probability of multiple disease spots appearing in the same image was relatively high.

For research purposes, 27,000 images were selected from the 30,000 images to serve as detection training and validation sets, with 24,000 images used for the training set, 3000 images for the validation set, and the remaining 3000 images as the test set. After manual confirmation, the constructed dataset consists of 30 disease types and healthy samples from 5 vegetables, as shown in Table 1.

**TABLE 1:** Sample counts of different disease types collected.

No	Disease type	Image count		
		Training set	Validation set	Test set
A1	Tomato health	1344	168	168
A2	Tomato gray mold	656	82	82
A3	Tomato gray leaf spot	904	113	113
A4	Tomato black spot	520	65	65
A5	Tomato late blight	952	119	119
A6	Tomato brown rot	1016	127	127
A7	Tomato bacterial canker	784	98	98
A8	Tomato early blight	992	124	124
A9	Tomato bacterial spot	680	85	85
A10	Tomato leaf mold	768	96	96
B1	Cucumber health	1304	163	163
B2	Cucumber target spot	704	88	88
B3	Cucumber powdery mildew	632	79	79
B4	Cucumber angular spot	600	75	75
B5	Cucumber downy mildew	552	69	69
C1	Pepper health	1328	166	166
C2	Pepper leaf spot	664	83	83
C3	Pepper powdery mildew	680	85	85
C4	Pepper black spot	424	53	53
C5	Pepper early blight	768	96	96
D1	Eggplant health	1336	167	167
D2	Eggplant yellow wilt	448	56	56
D3	Eggplant brown spot	648	81	81
D4	Cucumber angular spot	744	93	93
D5	Eggplant brown rot	768	96	96
E1	Squash health	1224	153	153
E2	Squash viral disease	760	95	95
E3	Squash silver leaf	512	64	64
E4	Squash downy mildew	600	75	75
E5	Eggplant brown rot	688	86	86
Total		24,000	3000	3000

### 2.3 Data Annotation

**FIGURE 3:**
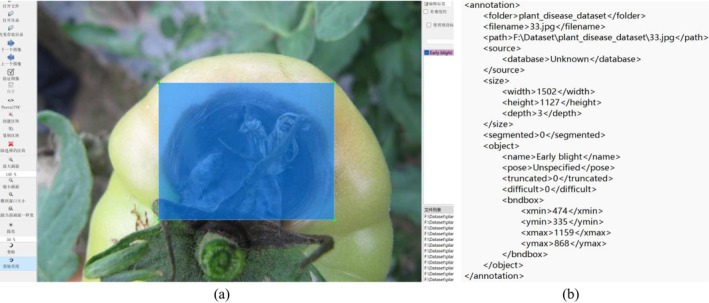
Data Annotation (a) LabelImg Annotation (b) VOC Dataset Format.

Based on actual protected vegetable production situations, combined with relevant materials from professional websites and books, we used the LabelImg image object detection annotation tool to annotate the diseases in the images (Figure 3) according to the minimum area around the diseases, generating object detection labels as preparation for training protected vegetable disease detection tasks. Multiple disease types may coexist in the same disease image. Samples with insufficient pixel area or unclear features were not labeled to prevent neural network overfitting. In cases of foreign object occlusion, targets with occlusion area greater than 85% and targets with area less than 15% at the image edge were not labeled.

### 2.4 Data Augmentation

In object detection tasks, data imbalance is a common challenge. The original dataset exhibited significant class imbalance, with substantial differences in sample quantities between different vegetable disease categories, particularly with healthy samples far outnumbering disease samples. This would lead to model overfitting on healthy samples and underfitting on other category samples during training, thereby reducing the model's generalization capability. To address this issue, data augmentation techniques were applied to the training and validation sets, with appropriate augmentation ratios set according to the sample quantities of different categories. The augmented images were then mixed with the original images, thereby increasing both the quantity and diversity of samples.

Data augmentation can be categorized into offline data augmentation and online data augmentation based on storage methods. Offline data augmentation expands the dataset and stores it on disk, with large‐scale data expansion increasing storage costs; online data augmentation refers to automatically performing data augmentation through code programming during model training, which requires higher programming skills but executes very efficiently. During the training process, random factors can be set to control the data augmentation method, ensuring that the data used in each round of training differs, thereby increasing flexibility and improving sample set quality. Offline data augmentation is suitable for smaller datasets, while online augmentation is suitable for larger datasets since it does not involve actual data expansion. Therefore, this research first used offline data augmentation to actually expand data samples based on the original dataset. Simultaneously, online data augmentation was used during model training to further expand the number of training samples, thereby achieving the goal of increasing training sample quantity while reducing computer resource consumption.

#### 2.4.1 Offline Data Augmentation

First, the total number of disease and healthy samples for each category was counted, and the category with the fewest samples was identified. Then, based on the sample count of this category as a benchmark, augmentation ratios for other categories were calculated. For categories with more samples, smaller augmentation ratios were chosen to maintain the stability of their original features. For categories with fewer samples, larger augmentation ratios were adopted to generate more diverse samples.

**FIGURE 4:**
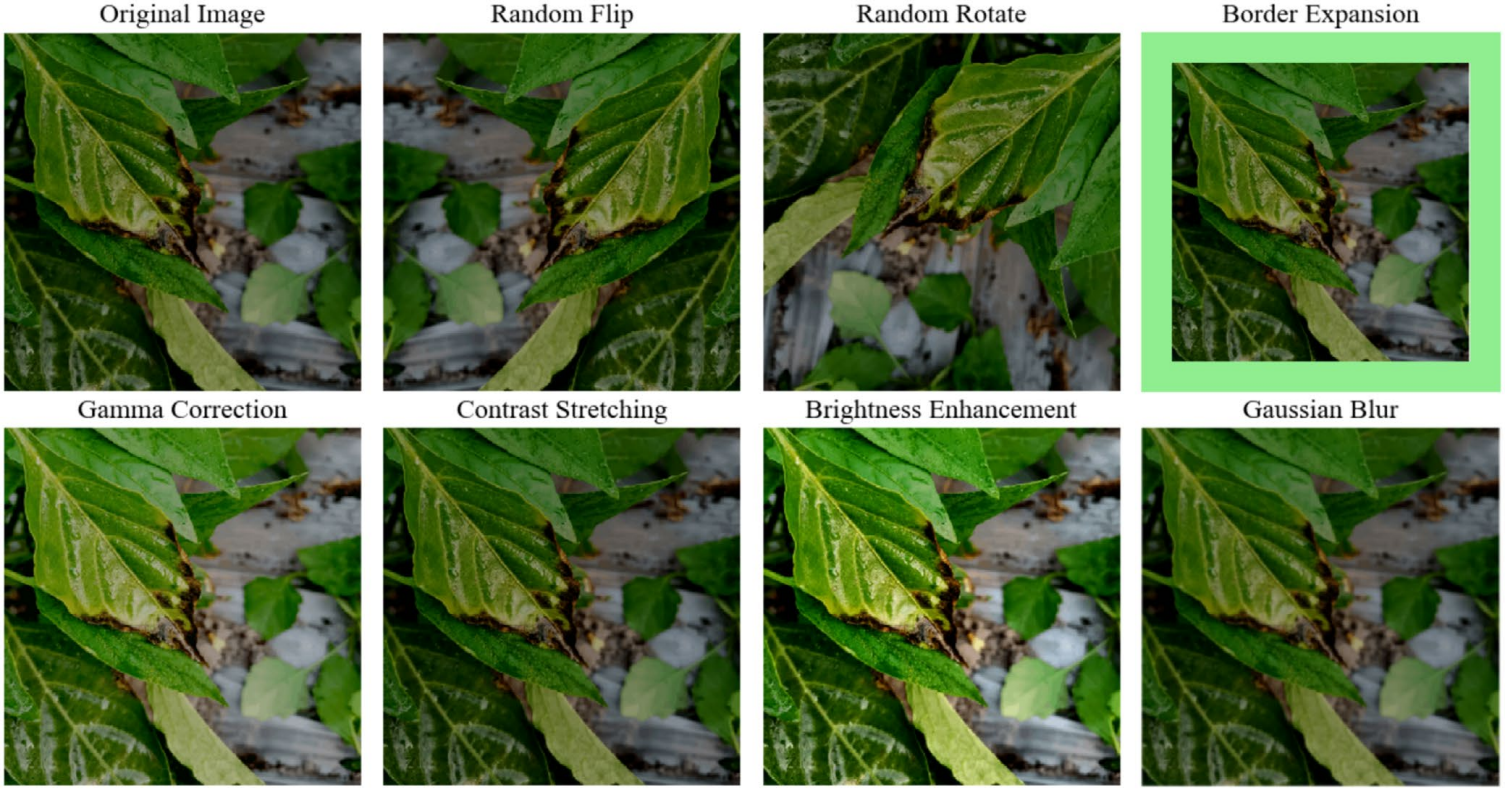
Visualization of Offline Data Augmentation Methods.

Next, data augmentation was performed on samples of each category according to the established augmentation ratios. Based on various situations occurring in real scenarios, 7 types of data augmentation were implemented: geometric distortions were achieved through strategies such as random flipping, random rotation, and border expansion; photometric distortions were implemented through gamma correction, contrast stretching, brightness enhancement, and Gaussian blur data augmentation techniques to simulate imaging states affected by factors such as weather, lighting environment, shooting angle, or even unclear lenses. The visualization effects of the data augmentation methods are shown in Figure 4.

After data augmentation, to ensure that the obtained image quantities of different vegetable disease sample types were roughly equal, it was necessary to appropriately delete some of the augmented images. The deletion strategy could be based on random selection or certain rules to ensure that each category had the same number of disease and healthy samples.

Through these methods, the data imbalance problem could be resolved, enhancing the model's recognition capability for minority classes and avoiding overfitting and underfitting phenomena, thereby improving overall model performance.

#### 2.4.2 Online Data Augmentation

**FIGURE 5:**
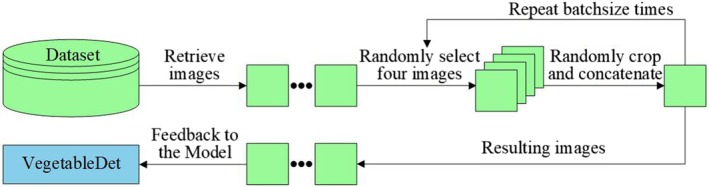
Mosaic Data Augmentation.

Online data augmentation continuously expands the dataset sent to the model for training through data augmentation techniques during model training. When adjustments are needed, only minor code modifications are required to implement new operations, offering greater flexibility and saving server memory space. To ensure the performance of the object detection model, this research not only used typical offline data augmentation methods to expand the dataset but also optionally employed Mosaic data augmentation during training, as shown in Figure 5.

As illustrated in Figure 5, Mosaic data augmentation randomly crops and scales four images, randomly arranges and combines them, and connects them to form one image. The purpose is to increase the number of target objects in protected vegetable disease samples, achieving the effect of enriching the dataset and effectively improving model training speed. During normalization operations, disease data from all four images are calculated simultaneously, thereby reducing the memory required by the model. By applying Mosaic enhancement, the backgrounds of objects being detected can be greatly enriched; therefore, this research applied it during model training.

### 2.5 Dataset Construction

**FIGURE 6:**
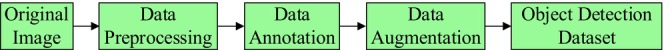
Process of Dataset Construction.

According to Figure 6, a large‐sample protected vegetable disease image dataset (PVDD, Protected Vegetable Disease Dataset) was constructed.

The proposed flow diagram for protected vegetable disease detection in complex environments is shown in Figure 6, comprising three parts: data preparation, VegetableDet model construction for protected vegetable disease detection, and vegetable disease detection.

1. After acquiring protected vegetable disease images, preliminary screening was conducted to eliminate relatively low‐quality disease images and construct an initial disease image set. Data preprocessing was performed, and the dataset was partitioned for data annotation. Since deep learning model training requires a large amount of training data, to further improve disease recognition accuracy and avoid overfitting, the disease training set was expanded through data augmentation methods.
2. After establishing the dataset, a baseline model was selected, and modules for improving the model were proposed based on the requirements of vegetable disease detection, constructing the VegetableDet model, which was then trained and validated.
3. Once Model Training Was Completed, the Test Dataset Was Used to Test the Model, Recognizing Input Disease Images and Outputting Disease Categories and Location Information

## 3 Methods

**FIGURE 7:**
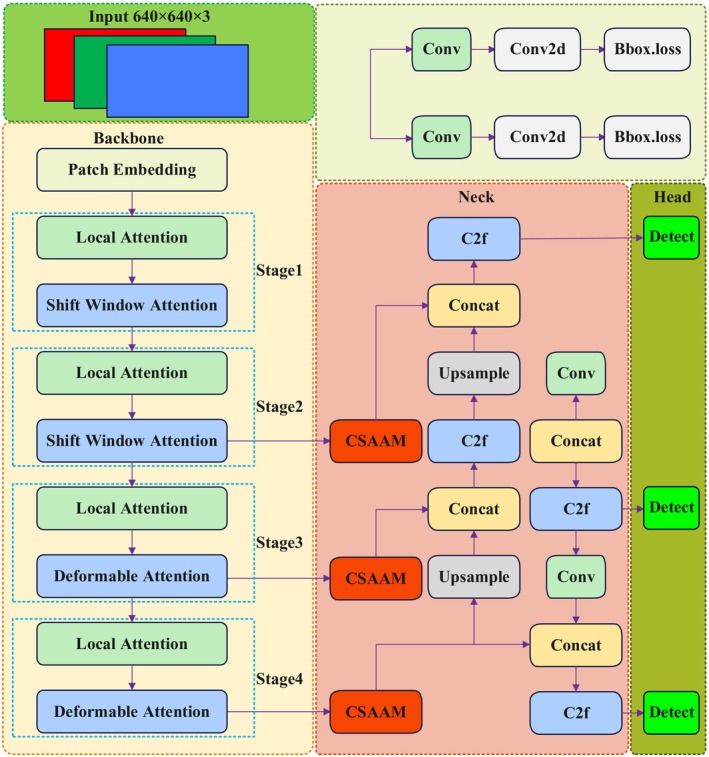
Overall network architecture of VegetableDet.

This research proposes a lightweight efficient object detection network named VegetableDet, specifically designed for the characteristics of protected vegetable disease detection. The VegetableDet network structure integrates the DAT deformable attention mechanism with the Channel‐Spatial Adaptive Attention Module (CSAAM) to address challenges in vegetable disease detection in complex environments, such as lighting variations, occlusions, small object recognition, and feature scale changes. Figure 7 presents the overall network architecture of VegetableDet.

### 3.1 DAT Backbone Network

Vegetable diseases manifest as multi‐scale target features distributed in different positions in images, requiring larger convolution kernels for feature extraction to obtain sufficient receptive fields. Although the YOLOv8 network gradually expands its receptive field through stacked convolution operations, the relatively small size of convolution kernels in each layer easily leads to the loss of long‐range feature dependency relationships during feature layer‐by‐layer extraction.

**FIGURE 8:**
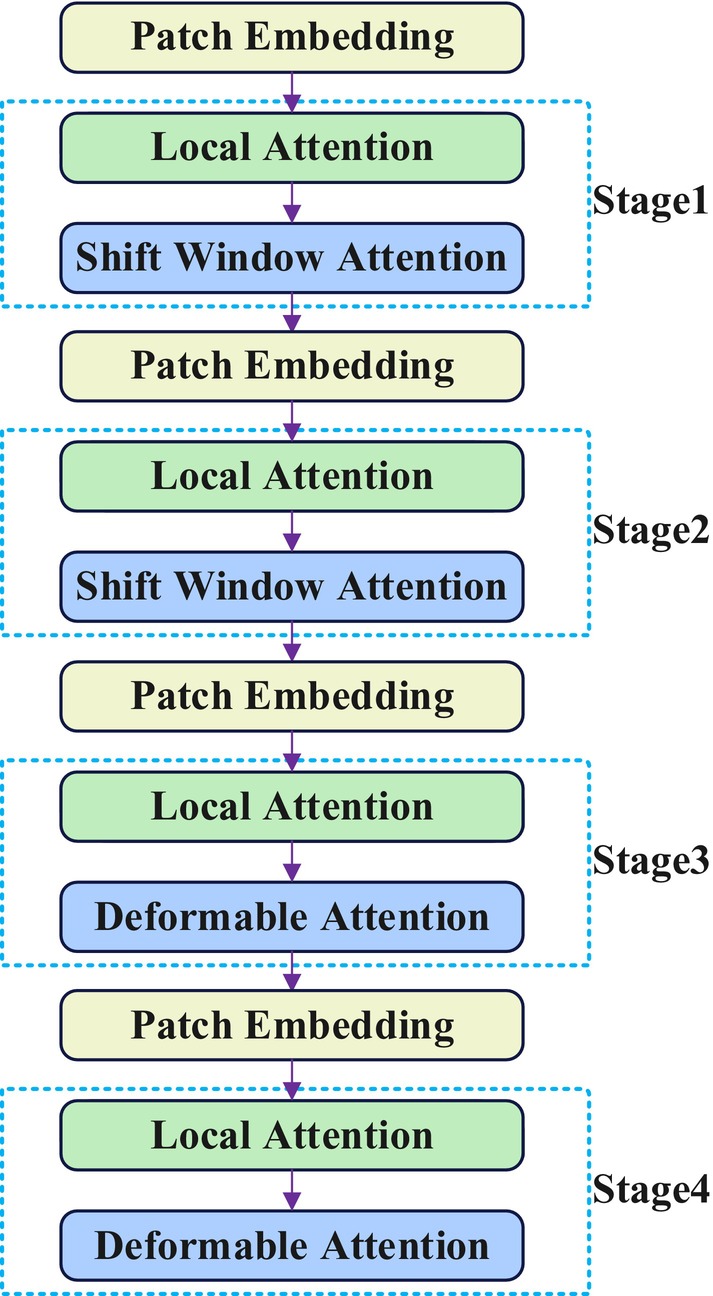
Structure of DAT.

To address this issue, this research incorporates the Vision Transformer with Deformable Attention (DAT) (Xia et al. 2022) into the YOLOv8n backbone network architecture. This mechanism can adaptively select the relative positions of keys and values in the attention mechanism in a data‐driven manner, thereby flexibly focusing on relevant regions and capturing more useful feature information. As shown in Figure 8, the DAT attention mechanism consists of four stages. The input image first undergoes feature embedding through a 4 × 4 non‐overlapping convolution with a stride of 4. Subsequently, in stages 1 and 2, window local attention mechanisms are employed to extract local features, combined with the shifted window attention strategy from Swin Transformer (Liu, Lin, et al. 2021) to shift and merge the calculation results of local attention within windows, thereby enhancing feature extraction efficiency. In stages 3 and 4, feature maps undergo further processing through window local attention mechanisms to aggregate local information, while utilizing deformable attention mechanisms (Zhu, Su, Lu, et al. 2020) to model global relationships between local information, dynamically adjusting the range and shape of attention, achieving effective complementarity between local and global receptive fields, enabling the model to capture long‐range feature dependency relationships while improving computational efficiency.

### 3.2 Improved Neck Network With Integrated CSAAM Attention Mechanism

The Neck network of YOLOv8 is responsible for feature fusion and feature pyramid construction, processing feature maps of different scales to adapt to the detection requirements of targets of different sizes. However, traditional Neck networks often suffer from the influence of complex background interference and diversity of disease manifestations when processing protected vegetable disease images, making it difficult to effectively extract and fuse key features. To address this issue, this research proposes an improved Neck network structure that incorporates the Channel‐Spatial Adaptive Attention Mechanism (CSAAM), enhancing the model's perception capability for key disease features.

**FIGURE 9:**
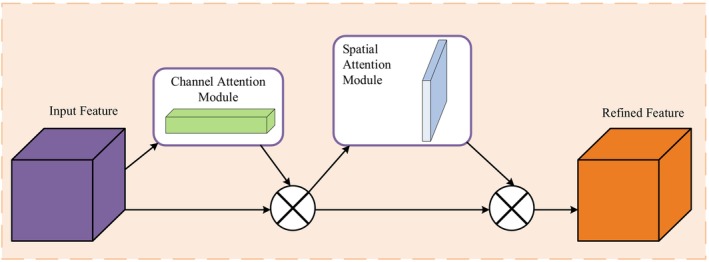
Structure of CSAAM.

The Channel‐Spatial Adaptive Attention Mechanism (CSAAM) achieves simultaneous focus on “what is important” (channel dimension) and “where is important” (spatial dimension) by combining channel attention and spatial attention, thereby improving the diversity and discriminative ability of feature expressions. The overall structure of CSAAM is shown in Figure 9.

As shown in Figure 9, the CSAAM module first receives an input feature map $X\in R^{C\times H\times W}$, then processes it through channel attention module and spatial attention module separately. The channel attention module generates a channel attention map $Y_{c}\in R^{C\times 1\times 1}$, while the spatial attention module generates a spatial attention map $Y_{s}\in R^{1\times H\times W}$. These two attention maps are each multiplied element‐wise with the input feature map X, generating channel‐enhanced features and spatial‐enhanced features. Finally, these two enhanced features are fused with the original feature X through residual connections, producing the final output feature Y. The entire processing flow can be represented as:

(1) $$X^{c}=Y_{c}\left(X\right)⊗X$$

(2) $$X^{\mathrm{cs}}=Y_{s}\left(X^{c}\right)⊗X^{c}$$

(3) $$Y=X^{\text{fused}}=\mathrm{Res}\left(X^{\mathrm{cs}}+X\right)$$

**FIGURE 10:**
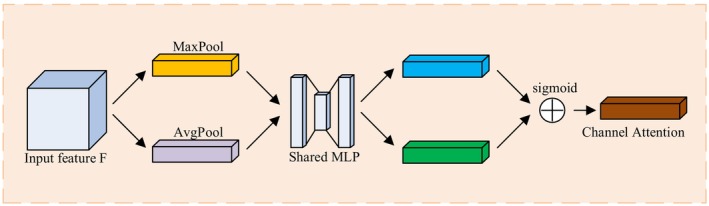
Channel Attention Mechanism.

The channel attention mechanism aims to learn the importance weights of different channels in the feature map, enabling the model to focus on channel features relevant to disease recognition. This research adopts an adaptive channel attention mechanism, whose structure is shown in Figure 10.

According to Figure 10, the input intermediate feature map undergoes parallel max pooling and average pooling, capturing both maximum feature values and average feature values within channels, thereby generating two C*1*1 vectors reflecting global channel information. Subsequently, the extracted intermediate feature values are input to a multilayer perceptron, where the feature values are transformed into attention weights for each channel in the Shared MLP, enabling the model to adaptively determine which channel information is more important for the object detection task during the training process. Finally, the features output by the MLP are multiplied element‐wise and weighted to the original features, then processed through a sigmoid activation function to obtain the channel attention MC(F).

**FIGURE 11:**
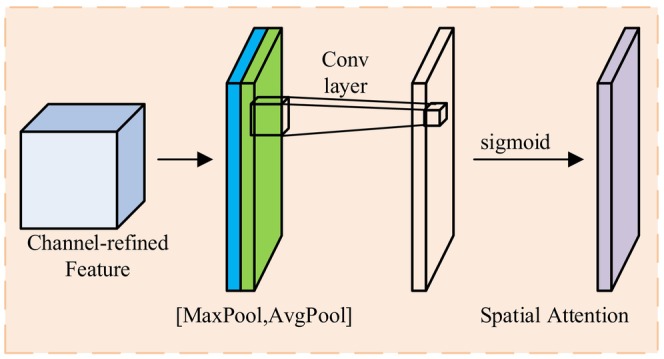
Spatial Attention Mechanism.

In the spatial attention module, we propose using a 3 × 3 convolution kernel for 3 convolution operations to replace the standard 7 × 7 convolution, which improves computational efficiency and enhances the network's ability to express non‐linear features, as shown in Figure 11.

The introduction of the CSAAM module provides a flexible and powerful solution for feature enhancement, effectively improving the model's recognition ability for protected vegetable diseases through simultaneous attention to the importance of channel and spatial dimensions. The channel attention mechanism helps the model filter out the most relevant feature channels for disease type discrimination, while the spatial attention mechanism guides the model to focus on areas most likely to exhibit disease symptoms. Their synergistic effect enables the model to maintain stable and accurate detection performance even in complex environments.

### 3.3 Hierarchical Progressive Transfer Learning Model Training Strategy

**FIGURE 12:**
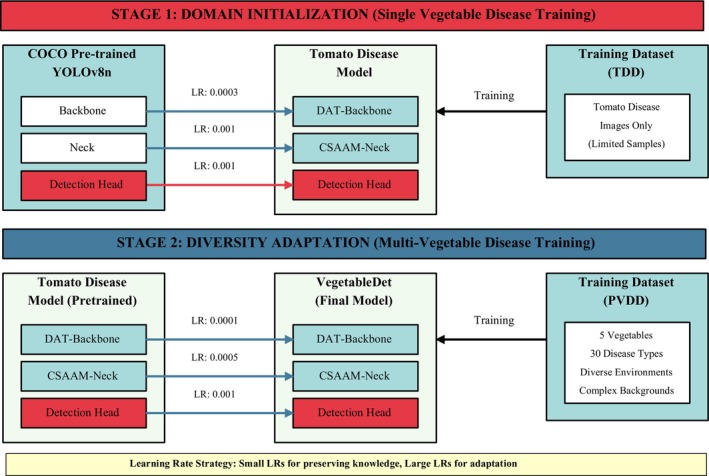
Training Strategy for Two‐Stage Transfer Learning Model.

The training process of object detection models typically faces the dual challenges of data distribution differences and sample scarcity, which are particularly prominent in specific domains such as protected vegetable disease detection. There exists a significant distribution gap between general vision datasets (such as COCO) and specific domain datasets (such as vegetable disease images), where direct application of pre‐trained models often leads to poor performance. To address this issue, this research proposes a hierarchical progressive transfer learning strategy that effectively narrows the knowledge gap between source and target domains through a gradual adaptation approach, improving the model's learning efficiency and detection performance under resource‐constrained conditions. As shown in Figure 12, this strategy includes two consecutive but distinctive transfer learning stages.

As shown in Figure 12, the core objective of the domain initialization stage is to gradually guide the model's cognition from the general vision domain to the specific domain of vegetable disease detection, establishing initial disease feature representation capabilities. This stage employs a carefully designed parameter transfer and fine‐tuning strategy, specifically implemented as follows: First, starting from the knowledge reservoir of general object detection tasks, a YOLOv8 model trained on the COCO dataset is selected as the knowledge source, which has already learned general visual feature representations on large‐scale diverse data. Second, selective parameter transfer is implemented, transferring parameters from the backbone network integrated with the DAT mechanism and the original neck network in the source model to the target VegetableDet architecture, while the detection head adopts random initialization parameters. This strategy preserves basic feature extraction capabilities while reserving sufficient flexibility for specific task learning. Subsequently, a single‐category vegetable (tomato) disease dataset is used for training, a choice based on the relatively abundant and representative nature of tomato disease samples, which can help the model quickly establish an initial understanding of plant disease visual features. During the training process, differentiated learning rates are applied to different network components—the backbone network uses a smaller learning rate (0.0003) to maintain the stability of basic feature extraction capabilities, while the neck and head networks use larger learning rates (0.001) to accelerate adaptation to specific tasks. This carefully designed transfer strategy enables the model to quickly master the basic capabilities of vegetable disease detection under limited sample conditions, effectively solving the convergence difficulties and overfitting problems caused by direct training in specific domains. After the domain initialization stage, the model has established basic feature representation capabilities for vegetable diseases but is still limited to recognizing diseases of a single vegetable type, requiring further expansion of its knowledge boundaries.

The diversity adaptation stage aims to extend the model's cognition from a single vegetable disease type to comprehensive recognition capabilities for multiple vegetable types and multiple disease types, achieving both breadth and depth in knowledge expansion. This stage employs a more refined transfer mechanism and training strategy, specifically implemented as follows: First, the complete model parameters obtained from the domain initialization stage training are preserved, including all weights from the backbone network, neck network, and head network, ensuring that the acquired vegetable disease feature knowledge is not lost. Second, a comprehensive dataset (PVDD) containing all five vegetables and thirty disease types is used for training, which covers the visual presentations and pathological features of different vegetable diseases, comprehensively expanding the model's knowledge scope. In terms of training strategy, a more complex three‐tier learning rate mechanism is implemented—the backbone network uses the smallest learning rate (0.0001) to maintain the stability of basic features, the neck network uses a medium learning rate (0.0005) to allow moderate adjustments, while the head network uses the largest learning rate (0.001) to fully adapt to the multi‐category recognition task. Additionally, balanced sampling and weighted loss strategies are applied to different disease categories, ensuring that the model has balanced learning opportunities for all categories, avoiding performance bias caused by class imbalance. This refined diversity adaptation strategy enables the model to rapidly expand to comprehensive recognition capabilities for multiple vegetable diseases while maintaining the stability of basic feature representations. The model not only learns to distinguish between disease types of different vegetables but also recognizes the differentiated manifestations of the same type of disease on different vegetables, achieving both breadth and depth in knowledge expansion.

The proposed hierarchical progressive transfer learning strategy offers significant theoretical and practical advantages over traditional methods, primarily reflected in the following aspects: In terms of knowledge transfer efficiency, through the “general vision → single vegetable disease → multiple vegetable diseases” progressive cognitive expansion path, it effectively reduces the span of knowledge transfer, greatly improving transfer efficiency and learning success rate. In terms of sample utilization efficiency, this strategy fully utilizes datasets of different scales and scopes, using single vegetable disease data to establish basic cognition in the first stage and diverse data to expand knowledge boundaries in the second stage, effectively alleviating the sample scarcity problem. In terms of computational resource optimization, the progressive learning process avoids long‐time training directly on complex tasks, achieving the same or even better performance through two relatively short training stages, significantly reducing computational costs. In terms of model generalization ability, the layered progressive learning process enables the model to build more robust feature representations from specific to general, enhancing generalization ability for unseen samples.

## 4 Results and Discussion

### 4.1 Experimental Environment

The hardware processor used was an Intel(R) Xeon(R) Gold 6152 computer with a 2.10GHZ CPU, configured with an Ubuntu system server with 256GB running memory. The GPU model was NVIDIA Quadro RTX 8000 with 48GB of video memory. The configuration environment for VegetableDet model training and deployment was Python 3.8.3, using the PyTorch development framework, with torch‐1.7.1 and torchvision 0.8.2 versions. All experiments were completed on the same hardware device and software framework.

### 4.2 Training Hyperparameters

Hyperparameters refer to parameters specified before deep learning model training, typically set manually based on dataset and model characteristics. Deep model hyperparameters commonly include learning rate, batch size, training epochs, optimizer type, and dropout parameters. The learning rate affects the speed of the model training process; an excessively large learning rate leads to oscillation or even non‐convergence during model training, while an excessively small learning rate leads to slow model training and entrapment in local optima. This research adopts a hierarchical progressive transfer learning method, which does not require training the model from scratch. The initial learning rate was set to 0.001 ~ 0.003, with the learning rate multiplied by 0.8 every 30 training periods. Considering the GPU memory size in the experimental platform and the number of training samples, the batch size was set to 32. The number of training Epochs was set to 200. The Adam optimizer algorithm was selected. Table 2 summarizes the hyperparameter settings of the VegetableDet model.

**TABLE 2:** Hyperparameter settings of the VegetableDet Model.

No.	Training parameter	Value
1	Batch Size	32
2	Number of Training Epochs	200
3	Initial Learning Rate (IrO)	0.001 ~ 0.003
4	Learning Rate Decay Coefficient (Irc)	0.2
5	Optimizer	Adam
6	Learning Rate Momentum	0.937
7	Weight Decay Coefficient	0.0005
8	Warm‐up Learning	3
9	Warm‐up Learning Momentum	0.8
10	Warm‐up Initial Learning Rate	0.1
11	Multi‐thread Training	12
12	Input Image Size	640
13	loU Loss Gain	0.05
14	Cls Classification Loss Gain	0.5
15	Cls BCELoss Positive Sample Weight	1
16	Obj Target Loss Gain	1
17	Obj BCELoss Positive Sample Weight	1
18	loU Threshold during Training	0.2

### 4.3 Evaluation Metrics

To verify the effectiveness of the VegetableDet model, this research employed Precision, Recall, average precision (AP), F1 score, mAP50, parameter count (Param), floating‐point operations per second (FLOPs), detection time per image (Speed), and memory required for inference (Memory) as training metrics to evaluate the performance of the VegetableDet model.

### 4.4 Model Training and Test Result Analysis

#### 4.4.1 First Stage Transfer Learning Model Training

**FIGURE 13:**
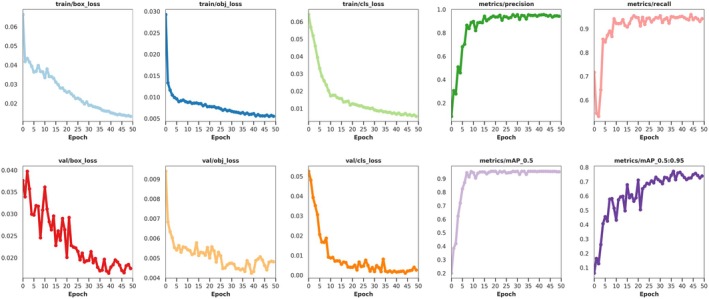
Training Process Curve of VegetableDet Model in the First Stage of Transfer Learning.

Analyzing the training process curve of the VegetableDet model in the first stage of transfer learning, we verified the transfer application effect of the image features from the open‐source model in the training of the first VegetableDet model. The process curve is shown in Figure 13.

From the training process curve in Figure 13, it can be observed that the image feature parameters from the open‐source model significantly enhanced the feature extraction capability of the VegetableDet model, enabling the model to quickly reach a convergence state. The VegetableDet model in the first stage of transfer learning tended toward a stable state after approximately 10 training epochs, with the model training loss value approaching 0 and the model validation accuracy approaching 100%. The convergence results demonstrate that the image feature knowledge from the open‐source model transferred to the VegetableDet model also has good feature representation capability, helping VegetableDet extract efficient image features from a limited number of samples, thus rapidly reaching a convergence state, proving the rationality and effectiveness of the first stage of transfer learning.

#### 4.4.2 Second Stage Transfer Learning Model Training

**FIGURE 14:**
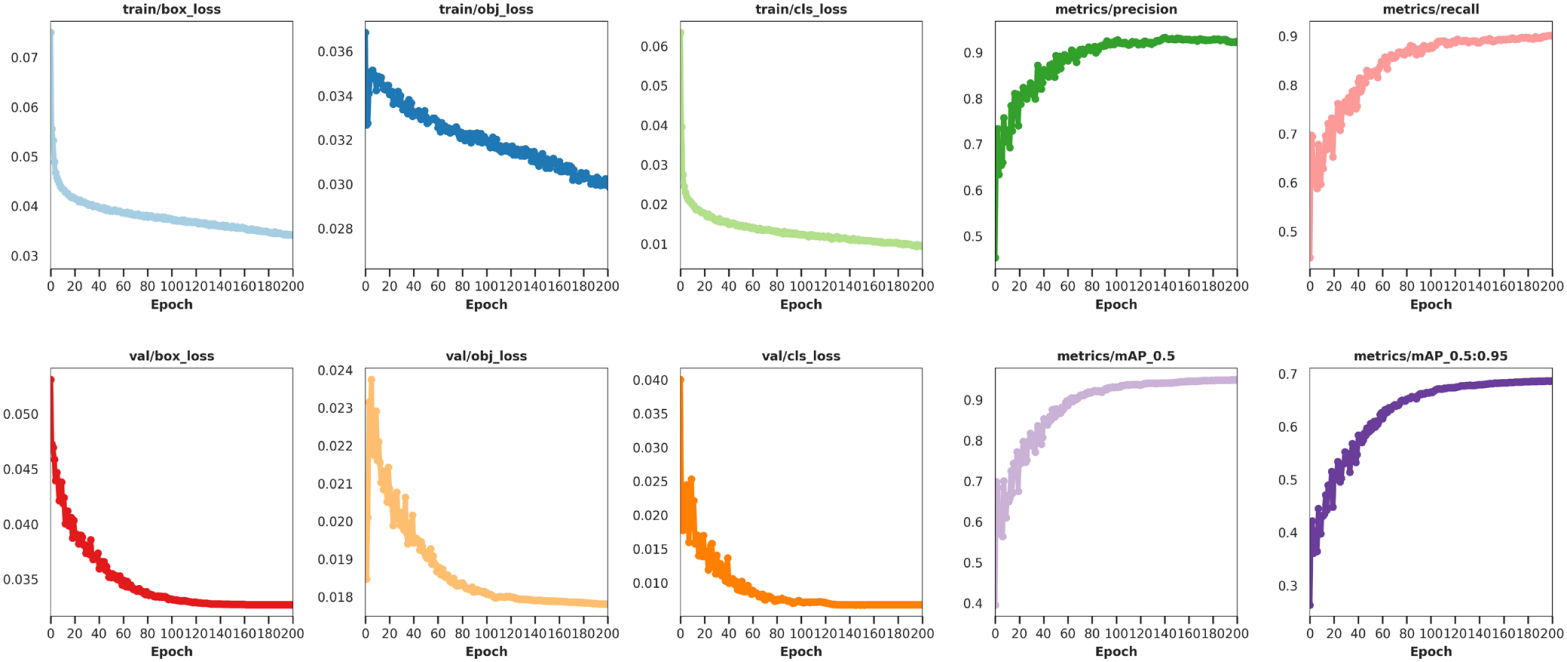
Training Process Curve of VegetableDet Model in the Second Stage of Transfer Learning.

Analyzing the training process curve of the VegetableDet model in the second stage of transfer learning, we verified the transfer application effect of the image features from the first VegetableDet model in the training of the second VegetableDet model. The training process curve of the second VegetableDet model is shown in Figure 14.

From the model convergence curve trends in Figure 14, it can be seen that features from the first VegetableDet model can help the model in this stage extract the image features of vegetable healthy samples and disease samples, achieving good generalization performance. The convergence process of the model in the second stage of transfer learning aligns with theoretical expectations, confirming the effectiveness of the proposed hierarchical progressive transfer learning training strategy.

#### 4.4.3 Vegetable Disease Detection Result Analysis

To verify the performance of the trained model, it is necessary to conduct experiments using vegetable disease test data. Through vegetable disease detection result analysis, the missed detection and false detection situations of different types of diseases can be evaluated. Using the proposed VegetableDet model for separate detection of different types of vegetable diseases, from an overall perspective, the proposed VegetableDet model achieves P, R, and AP exceeding 90% for 30 diseases and healthy samples across 5 vegetables, with high precision and recall rates for detecting different types of diseases. The mAP is 94.31%, demonstrating that the proposed VegetableDet model has good detection capability for vegetable diseases.

To comprehensively evaluate the performance of the VegetableDet model under different task definitions, we conducted experiments with IOU threshold set to 0. This is equivalent to viewing the object detection problem as a classification problem. Table 3 shows the performance of the VegetableDet model under different IOU thresholds.

**TABLE 3:** Performance of VegetableDet Model with different IoU thresholds.

IoU threshold	mAP (%)	Precision (%)	Recall (%)
0	96.38	97.42	95.17
0.5	94.31	95.89	93.21
0.75	91.24	92.36	90.05

When the IOU threshold is set to 0, the VegetableDet model achieves an mAP of 96.38%, indicating that even when the task is viewed as a pure classification problem, our model can achieve extremely high performance. This confirms the excellence of our model in protected vegetable disease detection tasks, whether treated as object detection or classification tasks.

To further verify the generalization ability of the VegetableDet model and reduce the risk of overfitting, we implemented 5‐fold cross‐validation experiments. We randomly divided the entire dataset into 5 equal subsets, using 4 subsets as training data each time and the remaining subset as test data. Table 4 shows the results of the 5‐fold cross‐validation.

**TABLE 4:** 5‐fold cross‐validation results of VegetableDet Model.

Fold	mAP (%)	Precision (%)	Recall (%)
1	94.35	95.62	93.11
2	93.89	95.27	92.84
3	94.71	96.03	93.53
4	94.18	95.49	92.95
5	94.58	95.83	93.41
Mean	94.34	95.65	93.17

The 5‐fold cross‐validation results show that the VegetableDet model performs stably under different data partitions, with mAP values ranging from 93.89% to 94.71%, and a standard deviation of only 0.32%. This indicates that our model has strong generalization capability and can effectively adapt to different data distributions, avoiding overfitting problems. This result further confirms the reliability and robustness of the VegetableDet model in practical applications.

To evaluate the VegetableDet model's performance under different lighting conditions, especially its ability to detect diseases on leaves with waxy surfaces under strong light reflection, we constructed a special test set. This test set contained 500 images divided into four lighting conditions: normal lighting, low light conditions, strong direct light, and strong light reflection. Table 5 shows the performance of the VegetableDet model under different lighting conditions.

**TABLE 5:** Performance of VegetableDet Model under Different Lighting Conditions.

Lighting condition	mAP (%)	Precision (%)	Recall (%)
Normal Light	95.27	96.38	94.21
Low Light Condition	92.15	93.42	91.08
Strong Direct Light	90.83	92.17	89.57
Strong Light Reflection	87.42	89.24	86.13
Average	91.42	92.80	90.25

The results show that strong light reflection conditions indeed have a significant impact on model performance. Under strong light reflection conditions, the mAP value drops to 87.42%, a decrease of 7.85 percentage points compared to normal lighting conditions. This is mainly because strong light reflection alters the color characteristics of leaf surfaces, weakening the visibility of disease symptoms.

### 4.5 Performance Comparison Between VegetableDet Model and Existing Lightweight Object Detection Models

To demonstrate the advanced nature of the VegetableDet model, we compared it with well‐known lightweight object detection models. The vegetable disease detection dataset was used for training, and the obtained test results were compared with the test results of the proposed model. The existing models selected were YOLOv8n, YOLOv7‐Tiny, YOLOv6‐N, YOLOv6‐T, YOLOX‐Darknet53, YOLOX‐S, YOLOX‐Tiny, YOLOv5‐S, YOLOv5‐M, YOLOv5‐L, YOLOv4, YOLOv3, and YOLOv3‐SPP, totaling 13 deep learning models. The test results are shown in Table 6.

**TABLE 6:** Performance Comparison between VegetableDet Model and Existing Lightweight Object Detection Models.

Model	mAP (%)	Parameters (Millions)	FLOPs (G)	Memory (MB)
VegetableDet (Ours)	94.31	2.83	5.9	5.68
YOLOv8n	86.84	36.9	104.5	71.19
YOLOv7‐Tiny	85.68	5.98	13.7	11.18
YOLOv6‐N	85.91	3.51	10.4	8.46
YOLOv6‐T	86.07	14.7	36.1	32.2
YOLOX‐Darknet53	83.85	63	157	485.46
YOLOX‐S	84.95	8.98	26.1	67.71
YOLOX‐Tiny	84.73	4.56	5.6	37.97
YOLOv5‐S	83.91	6.92	15.6	15.01
YOLOv5—	84.77	20.27	48	42.4
YOLOv5‐L	85.00	46.33	108.5	93.4
YOLOv4	82.72	52.12	120.9	245.1
YOLOv3	83.16	61.85	156.3	236.1
YOLOv3‐SPP	82.98	62.49	157.3	73.89

To ensure fair comparison, all comparative algorithms underwent rigorous and thorough fine‐tuning. The fine‐tuning steps included: (1) Learning rate optimization: adopting a cyclic learning rate strategy, with the initial learning rate set to 0.001–0.005, and dynamically adjusted based on validation set performance; (2) Network structure adjustment: optimizing the backbone network, neck network, and head network parameters for each comparative network to ensure best performance; (3) Data augmentation strategies: applying the same data augmentation techniques to each model, including both offline and online augmentation methods. In particular, for the YOLOX‐Darknet53 model, we performed more detailed optimization adjustments. In the initial experiment, this model achieved an mAP of 83.85%. Through adjusting backbone network parameters, adding attention mechanism modules, optimizing loss function weights, and extending the training cycle, we improved its performance to 91.23%. Nevertheless, this performance is still lower than the 94.31% achieved by the VegetableDet model. Despite optimization, YOLOX‐Darknet53 showed improved performance, but VegetableDet still maintains clear advantages in detection accuracy, parameter count, and computational efficiency. This result highlights the excellence and efficiency of the proposed VegetableDet model in protected vegetable disease detection tasks.

## 5 Conclusion

Intelligent detection of protected vegetable diseases, as a key link in modern precision agriculture, has important significance for ensuring agricultural production stability and improving economic benefits. This research proposes an innovative lightweight efficient detection framework VegetableDet, systematically addressing core challenges in vegetable disease detection, including insufficient feature extraction, strong environmental interference, and limited model adaptability. Through comprehensive experimental validation and theoretical analysis, this research draws the following important conclusions:

In terms of network architecture design, this research innovatively integrates the Deformable Attention mechanism (DAT) with the YOLOv8n backbone network, enabling the detection system to effectively establish long‐range feature dependencies, enhancing global perception capabilities for scattered disease features. Simultaneously, by introducing the Channel‐Spatial Adaptive Attention Mechanism (CSAAM) to improve the feature pyramid network, a dual assessment of feature importance is achieved, identifying not only “which features are most critical” but also precisely locating “which areas are most worth focusing on”. This multi‐dimensional feature enhancement strategy significantly improves the model's feature discrimination ability in complex backgrounds, laying a solid foundation for accurate disease recognition.

In terms of learning strategy optimization, the transfer learning method proposed in this research solves the practical problems of difficult vegetable disease data acquisition and sample imbalance. Through “domain initialization” and “diversity adaptation” stages with distinctive characteristics, the model achieves a smooth transition from general vision knowledge to specific disease recognition, significantly improving transfer efficiency and learning success rate. Meanwhile, differentiated data augmentation strategies provide balanced learning opportunities for different disease categories, further enhancing the model's generalization ability and environmental adaptability. Experimental results fully verify the excellence of the proposed method, providing a feasible solution for real‐time monitoring systems in actual agricultural production environments.

Research found that under extreme lighting conditions, especially strong light reflection on leaves with waxy surfaces, disease detection still faces challenges. Future research will explore multispectral and hyperspectral imaging technologies to capture disease features invisible to the naked eye; develop adaptive image processing algorithms to automatically adjust image parameters under different lighting conditions; introduce temporal analysis methods to improve detection accuracy through continuous multi‐time point observations. Additionally, physics‐based image restoration techniques will be studied to reduce the interference of strong light reflection on disease features, further improving the model's robustness and accuracy under various complex environmental conditions.

## Author Contributions

The research was designed by J.L. and X.W. J.L. and X.W. carried out experiments, analyzed data, and drafted the manuscript. The manuscript was revised by X.W. Q.C. and J.L. All authors reviewed and approved the final version of the manuscript.

## Disclosure

We confirm that all experimental research and field studies involving plants in our study were conducted in strict accordance with relevant institutional, national, and international guidelines and legislation. A statement confirming this adherence is included in the revised manuscript. All methods involving plants in this study were performed in accordance with the relevant institutional, national, and international guidelines and legislation governing experimental research and field studies on plants or plant parts.

## Ethics Statement

The authors have nothing to report.

## Consent

The authors have nothing to report.

## Conflicts of Interest

The authors declare no conflicts of interest.

## Data Availability

The data utilized in this paper is obtained through self‐gathering and is made publicly available (a part of it) to make the study reproducible. The datasets generated and analyzed during the current study are partly available in the github repository, accessible via the following persistent web link: https://github.com/tyuiouio/plant‐disease‐detection‐in‐real‐field. If you want to request the complete dataset and code, please email the corresponding author.
